# 

**Published:** 1912-02

**Authors:** 


### CONTENTS.

ORIGINAL COMMUNICATIONS: —

The Prevention of Post-Operative Gynecological Psychoses. By Herbert P. Cole, M. D., Mobile, Ala. .......... 575

Puerperal Eclampsia. By J. S. Horseley, West Point, Ga. .......... 578

Dysmenorrhea of Nasal Origin—With Report of a Case. By Thos. F. Taylor, M. D., Tuskegee, Ala. .......... 584

Importance of County and City Boards of Health. By H. W. Terrell .......... 588

Lobar Pneumonia. By J. P. Liles M. D., Roanoke, Ala. .......... 592

SELECTIONS AND ABSTRACTS .......... 598

EDITORIALS .......... 597

